# Supplementary material for: Longitudinal Genome-Wide Association Study of Cognitive Impairment after Subarachnoid Hemorrhage
Source: Biomedicines. 2024 Jun 22;12(7):1387. doi: 10.3390/biomedicines12071387 (PMC11275094; doi:10.3390/biomedicines12071387)
Supplement: Supplementary file 1 [file biomedicines-12-01387-s001.zip › biomedicines-3043896-supplementary.pdf]

SUPPLEMENTAL DATA

Supplementary Note.
Koran version of MMSE

The Koran version of the MMSE (K-MMSE) was used to evaluate cognitive function. K-MMSE involved 30 questions regarding time and place orientation (10 points), memory (registration 3 points and recall 3 points), attentions and calculation (5 points), language (8 points), and visuospatial function (1 point) (Reference 1 below). The test was usually completed within 10 minutes. The first K-MMSE test was performed 6 months after SAH ictus by referral to a neurologist or psychiatrist. Thereafter, the test was repeated every year regardless of the results of first K-MMSE [1]. An MMSE score less than 27 indicates cognitive impairment (3). Eagles et al. chose the MMSE score of less than 27 as a cut-point to ensure adequate sensitivity of cognitive impairment for those with college-educated individuals referring to previous studies [4, 5].

References

1. Park H, Jeon S, Lee J, et al. Korean Version of the Mini-Mental State Examination Using Smartphone: A Validation Study. Telemed J E Health. 2017;23(10):815-821.
2. Han SW, Kim BJ, Kim TY, et al. Association of haptoglobin phenotype with neurological and cognitive outcomes in patients with subarachnoid hemorrhage. Front Aging Neurosci. 2022;14:819628.
3. Eagles ME, Tso MK, Macdonald RL. Cognitive Impairment, Functional Outcome, and Delayed Cerebral Ischemia After Aneurysmal Subarachnoid Hemorrhage. World Neurosurg. 2019:S1878-S18750(19)30020-8.
4. O'Bryant SE, Humphreys JD, Smith GE, et al. Detecting dementia with the mini-mental state examination in highly educated individuals. Arch Neurol. 2008;65:963-967.
5. Franceschini M, Massimiani, MP, Paravati S, et al. Return to work: a cut-off of FIM gain with Montebello rehabilitation factor score in order to identify predictive factors in subjects with acquired brain injury. PLoS One. 2016;11:e0165165.

Supplementary Figure S1. Flow chart of included and excluded patients.

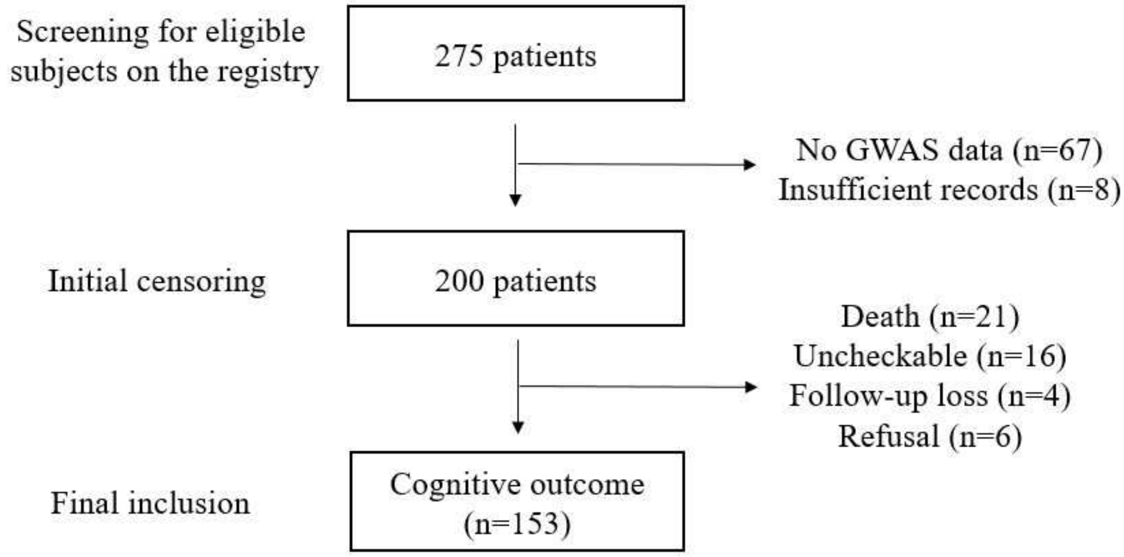

Supplementary Figure S2. Western blotting analysis of haptoglobin (Hp) phenotypes. Hp alleles were confirmed using polyacrylamide gel electrophoresis, followed by immunoblotting to identify α1 and α2 chains referring to our previous protocols13,19 : Three types of haptoglobin were classified, and then western blotting was performed again targeting only Hp2-1. For analysis, a 1:75 dilution of serum was made by adding a 1 μl of serum to 74 μl of phosphatebuffered saline. Samples were prepared by mixing a serum diluent with an equal volume of 2xSDS sample buffer (Bio-Rad, CA, USA) and boiled at 95 °C for 8 min. After boiling, 10 μl of sample was loaded on 15 % polyacrylamide gel and electrophoresed for 150 min at 100 V (BioRad, CA, USA). After transfer, the membranes were blocked with 5 % BSA in TBST (10 mM Tris-HCl pH8.0, 150 mM NaCl) including 0.01 % Tween-20 for 1 hr. The membranes were incubated overnight with polyclonal rabbit anti-human haptoglobin antibody (Dako, Denmark) diluted 1:10,000 in blocking buffer at 4 °C. After three washes with TBST, the membranes were incubated with horseradish peroxidase (HRP)-conjugated goat anti-rabbit IgG (Abcam, UK) in a 1:10,000 for 1 hr at room temperature. Following a final washing steps, HRP substrate (Thermo, USA) was added to a membrane and chemiluminescence was detected using X-ray film (Kodak, USA). To determine the polymeric composition of Hp2-1 based on molecular size, additional immunoprecipitation was carried out with anti-Hp antibody. To immunoprecpitate Hp, 0.3 mg/ml of the serum from patient with Hp2-1 was incubated overnight with anti-Hp antibody at 4 °C. The immune complexes were precipitated with protein A/G Sepharose (Santa Cruz, USA) and analyzed by western blotting. Anti-albumin antibody (Abcam, UK) was used as a loading control. The band intensities of α1- and α2- chain were analyzed using ImageJ software (Version 1.49v, National Institutes of Health, USA).

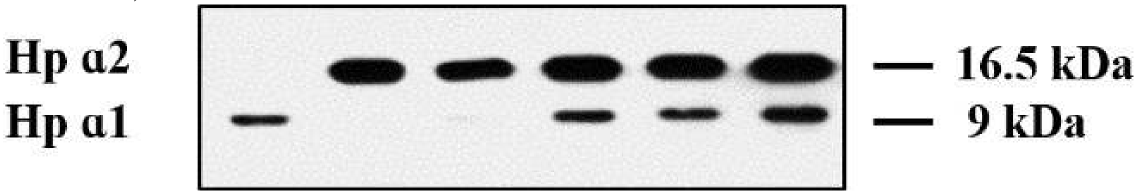

Supplementary Figure S3. Regional association plots of four loci ± 500 kb including rs138753053 (*PDCD6IP-LOC101928135*), rs56823384 (*LINC00499*), rs145397166 (*CASC15*), and rs10503670 (*LPL-SLC18A1*): that showed a genome-wide association with cognitive impairment following subarachnoid hemorrhage. Triangles and reverse triangles indicate positive and negative effect sizes, respectively, and each color shows pairwise linkage disequilibrium. The purple up-triangle indicates the top SNP in each region. Other colors indicate pairwise linkage disequilibrium (LD, r^2): navy, 0.0 – 0.2; green, 0.2 – 0.4; sky-blue, 0.4 – 0.6; orange, 0.6 – 0.8; red, 0.8 – 1.0.

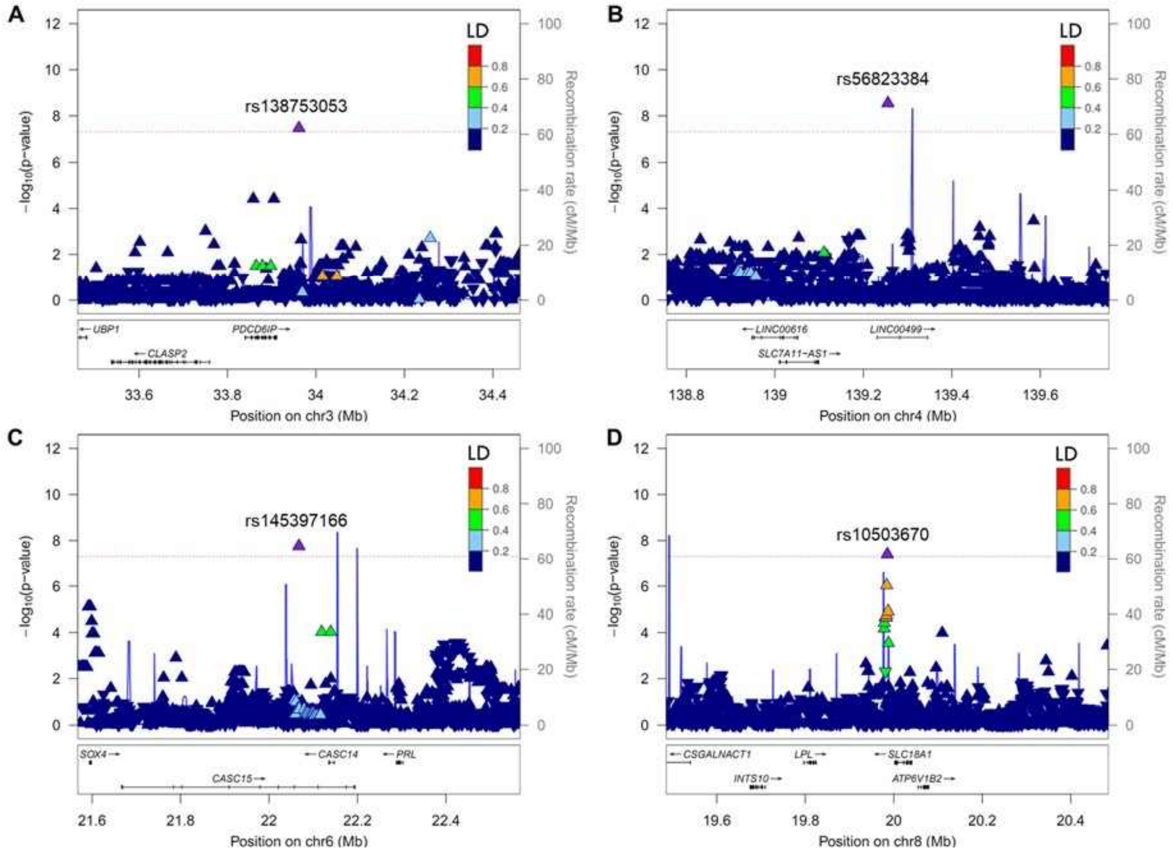

Supplementary Table S1. Baseline characteristics of haptoglobin phenotypes

Variables	Cognitive impairment (N = 65)	Non-cognitive impairment (N = 88)	HR (95% CI)[a]	P[a]
Hp 1-1	1 (1.5%)	13 (14.8%)	Reference	
Hp 2-1	18 (27.7%)	39 (44.3%)	6.58 (0.88-49.36)	0.067
Hp 2-2	46 (70.8%)	36 (40.9%)	9.82 (1.35-71.30)	0.024

Data are described with the number of subjects (percentage).

[a] Hazard ratio (HR), 95% confidence intervals (CI), and p-value were from Cox-proportional hazard regression model.

Supplementary Table S2. Subgroup analysis of the five genome-wide significant single nucleotide polymorphisms (SNPs) that have been associated with developing incidental events of the cognitive impairment after subarachnoid hemorrhage according to haptoglobin (Hp) subtypes.

SNP	M/m[a]	Hp2-1[b]			Hp2-2[b]		
		HR	95% CI	*P*	HR	95% CI	*P*
rs138753053	G/A	24.77	3.25-188.79	0.002	141.64	11.42-1756.64	1.2×10^{-4}
rs56823384	T/C	24.74	5.50-111.38	2.9×10^{-5}	8.71	2.71-28.03	2.8×10^{-4}
rs145397166	C/G	12.51	1.82-85.82	0.01	10.02	3.60-27.85	1.0×10^{-5}
rs10503670	G/A	2.60	1.27-5.30	0.009	3.62	2.19-6.00	5.5×10^{-7}
rs76507772	A/C	7.41	1.70-32.21	0.008	5.70	2.67-12.17	6.7×10^{-6}

[a] M/m indicates a major and minor allele, respectively.

[b] Hazard ratio (HR), 95% confidence intervals (CI), and p-value were from Cox-proportional hazard regression model.

Supplementary Table S3. Weighted polygenic risk score model to predict the incidental events of cognitive impairment following subarachnoid hemorrhage.

Risk model[a]	Cognitive impairment, N (%)	Non-cognitive impairment, N (%)	HR (95 CI)[b]	*P*[b]	AUROC[c]	Sensitivity[c]	Specificity[c]
All subjects							
T1: < 1.06	26 (40.0)	75 (85.2)	Reference				
T2: 1.06-2.12	16 (24.6)	9 (10.2)	5.81 (2.93-11.48)	4.4×10^{-7}		0.600	0.852
T3: 2.12 <	23 (35.4)	6 (4.6)	18.22 (9.12-36.41)	2.1×10^{-16}	0.739	0.354	0.955
Hp2-1							
T1: < 1.06	6 (33.3)	34 (87.2)	Reference				
T2: 1.06-2.12	6 (33.3)	3 (7.7)	6.36 (1.63-24.60)	0.007		0.667	0.872
T3: 2.12 <	6 (33.3)	2 (5.1)	44.59 (8.61-231.08)	6.1×10^{-6}	0.774	0.333	0.949
Hp2-2	n=46	n=36					
T1: < 1.06	20 (43.5)	31 (86.1)	Reference				
T2: 1.06-2.12	10 (21.7)	5 (13.9)	7.86 (2.99-20,65)	2.9×10^{-5}		0.565	0.861
T3: 2.12 <	16 (34.8)	0 (0.0)	13.13 (5.99-28.78)	1.2×10^{-10}	0.737	0.348	1.000

[a] The patients were stratified into risk tertiles (low (T1), middle (T2), and high (T3) risk).
[b] Hazard ratio (HR), 95 confidence interval (CI), and p-value were estimated from multivariate Cox-proportional regression model.
[c] Predictability, sensitivity, and specificity were derived from the area under the receiver-operating characteristics curve (AUROC) analysis.
